# Melatonin plays a synergistic rather than a major role during osteogenic differentiation via MT2 in mouse mesenchymal stem cell line C3H10T1/2: Melatonin plays a synergistic role during osteogenic differentiation

**DOI:** 10.3724/abbs.2023095

**Published:** 2023-07-05

**Authors:** Le Wu, Ying Xu, Wenqi Sun, Jun Sun, Yong Chen, Lifeng Liu

**Affiliations:** 1 Department of Trauma Orthopaedics Shanghai East Hospital Tongji University School of Medicine Shanghai 200120 China; 2 State Key Laboratory of Molecular Biology National Center for Protein Science Shanghai Shanghai Institute of Biochemistry and Cell Biology Center for Excellence in Molecular Cell Science Chinese Academy of Sciences Shanghai 200031 China

Melatonin, a multifunctional hormone mainly synthesized and secreted by the pineal gland, is increased at night and decreased during the day [1]. Its secretion and rhythm are spontaneously reduced with aging, which is accompanied by an increase in the incidence of osteoporosis [2]. This phenomenon suggests that melatonin may play a major role in preserving bone mass. Age-related osteoporosis is a systemic skeletal disease characterized by low bone mass and microarchitectural deterioration of bone tissue, resulting in the increase of osteoclastogenesis and adipogenesis and the inadequateness of osteoblastogenesis [3]. Osteogenic differentiation, a process in which mesenchymal stem cells (MSCs) differentiate into preosteoblasts, immature osteoblasts, and finally mature osteoblasts, is critical for the deposition of mineralized matrix to facilitate the formation of bone. However, the role of melatonin and its regulatory mechanism during osteogenic differentiation remain elusive.

Runx2, a transcription regulator acting on the DNA runt domain, is an indispensable protein in osteogenic differentiation. During the differentiation of MSCs into osteoblasts, Runx2 is expressed slightly in undifferentiated MSCs and significantly in preosteoblasts and immature osteoblasts but sharply decreased in mature osteoblasts. Runx2 ^‒/‒^ mice showed a complete lack of ossification, and both intramembranous and endochondral ossification were completely blocked [4]. Furthermore, forced expression of Runx2 in nonosteoblastic cells induced the expressions of the principal osteoblast-specific genes [5]. All these results suggest that Runx2 may be a regulatory factor that makes cells have osteogenic characteristics during the differentiation of MSCs. Thus, Runx2 plays a pivotal role in bone formation and is a recognized marker of osteogenic differentiation.

Many studies have determined the effect of melatonin on Runx2 expression. For instance, in osteogenic culture medium, melatonin increased Runx2 expression, promoting osteogenic differentiation of MSCs or reducing the inhibitory action of ethanol, cadmium or glucocorticoids on osteogenic differentiation of MSCs [6]. However, in the absence of exogenous induction, the effect of melatonin on Runx2 expression remains unclear. Herein, we aim to determine the effect of melatonin at different concentrations on Runx2 expression and clarify its role and regulatory mechanism during osteogenic differentiation. Information garnered from this study could be helpful for further exploring adjuvant treatment of osteoporosis with melatonin.

To determine the effect of melatonin on Runx2 expression in the absence of exogenous induction, we incubated mouse mesenchymal stem cell line C3H10T1/2 in basal growth culture medium containing 1 nM–1 mM melatonin for 24 h. Western blot (WB) analysis and real-time quantitative polymerase chain reaction (RT-qPCR) confirmed that the expression of Runx2 at both the protein and mRNA levels was slightly increased by melatonin in a concentration-dependent manner ( Supplementary Figure S1), suggesting that the effect of melatonin alone on Runx2 expression is very weak in the absence of exogenous induction during osteogenic differentiation of MSCs. Thus, 1 mM melatonin was used in the subsequent experiments.

Bone morphogenetic protein 2 (BMP2), a member of the TGF-β family, was reported to be able to increase Runx2 expression and promote osteogenic differentiation of MSCs [7]. Since melatonin may be auxiliary to reducing the high expression of Runx2 and in the absence of exogenous induction, it may only slightly rather than significantly increase Runx2 expression. Therefore, we sought to investigate whether melatonin can reduce BMP2-induced increased Runx2 expression and promote osteogenic differentiation of MSCs. C3H10T1/2 cells were incubated in growth culture medium containing BMP2 at different concentrations for 72 h, and then WB analysis and RT-qPCR were used to analyze Runx2 expression at the protein and mRNA levels, respectively. We found that BMP2 significantly increased Runx2 expression in a dose-dependent manner ( Supplementary Figure S2A‒C). Based on the above results, 120 ng/mL BMP2 was used for the subsequent experiments.

To test whether melatonin is auxiliary to reducing BMP2-induced stimulatory effects, C3H10T1/2 cells were pretreated with 1 mM melatonin for 1 h before adding 120 ng/mL BMP2 for 72 h in growth culture medium. We found that although melatonin slightly or BMP2 evidently increased Runx2 expression at the protein and mRNA expression levels, pretreatment with melatonin significantly enhanced rather than reduced BMP2-induced increase in Runx2 expression ( Supplementary Figure S2D‒F). Additionally, to further determine whether melatonin enhances BMP2-induced promotion of osteogenic differentiation of MSCs, C3H10T1/2 cells were pretreated with 1 mM melatonin for 1 h before adding 120 ng/mL BMP2 for 2 or 4 weeks in osteogenic induction culture medium. Alkaline phosphatase (ALP) activity or alizarin red staining showed that although melatonin or BMP2 promoted osteogenic differentiation of MSCs, pretreatment with melatonin significantly enhanced rather than reduced BMP2-induced promotion of osteogenic differentiation ( Supplementary Figure S2G,H). These results demonstrate that the effect of melatonin alone on the osteogenic differentiation of MSCs is very weak in the absence of exogenous induction; therefore, when MSCs are subjected to a pro-osteogenic stimulus, melatonin plays a synergistic rather than a major role during osteogenic differentiation in mouse mesenchymal stem cell line C3H10T1/2.

Melatonin exerts its regulatory function mainly by binding to its two G protein-coupled receptors on the cell membrane: melatonin receptor 1A (MT1, encoded by MTNR1A) and melatonin receptor 1B (MT2, encoded by MTNR1B) [8]. To further verify whether the actions of melatonin on BMP2-induced stimulatory effects during osteogenic differentiation of MSCs are associated with MT1 and MT2, C3H10T1/2 cells were pretreated with 10 μM luzindole [9], a nonselective antagonist for MT1 and MT2, or 10 μM 4P-PDOT [9], a selective antagonist for MT2, for 1 h before adding 1 mM melatonin, and then 120 ng/mL BMP2 was added to the cells for 72 h in growth culture medium or for 2 or 4 weeks in osteogenic culture medium. We found that luzindole or 4P-PDOT could strikingly block the role of melatonin in enhancing BMP2-induced stimulatory effects on the protein and mRNA expression levels of Runx2 and osteogenic differentiation of MSCs ( Figure 1). These data indicate that the synergistic role of melatonin in enhancing BMP2-induced osteogenic effects during osteogenic differentiation of MSCs is to a great extent via MT2 rather than MT1.

**Figure 1:**
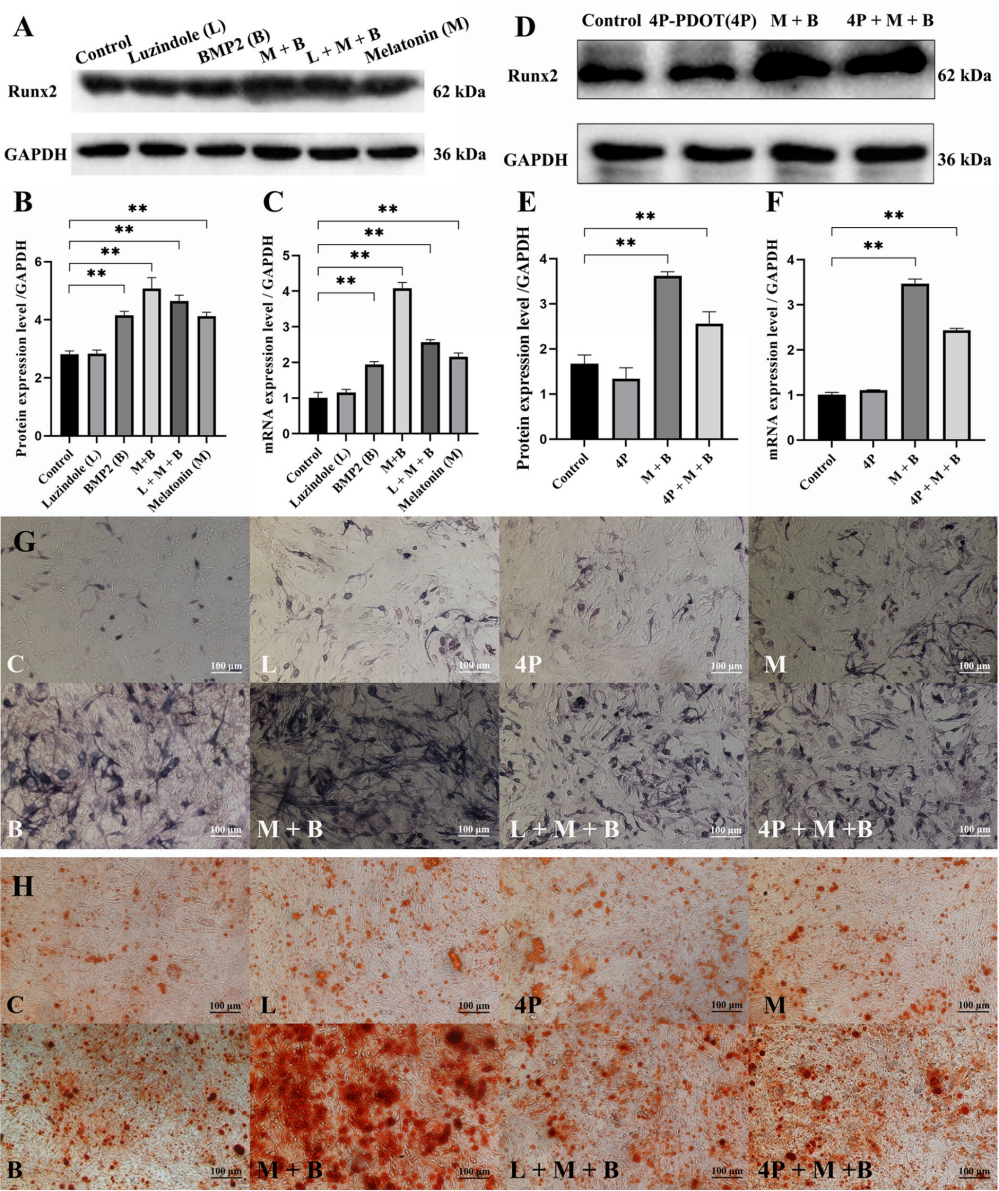
Figure 1 The synergistic effect of melatonin on BMP2-induced osteogenic differentiation of MSCs is associated with MT1 and MT2 (A) Western blot analysis was used to analyze Runx2 protein expression levels in C3H10T1/2 cells pretreated with 10 μM luzindole for 1 h, followed by the addition of 1 mM melatonin, and then cultured in growth medium with 120 ng/mL BMP2 for 72 h. (B) Quantification of the western blot in (A). (C) RT-qPCR was used to analyze the expression levels of the Runx2 gene in C3H10T1/2 cells pretreated with 10 μM luzindole for 1 h, followed by the addition of 1 mM melatonin, and then cultured in growth medium with 120 ng/mL BMP2 for 72 h. (D) Western blot analysis was used to analyze Runx2 protein expression levels in C3H10T1/2 cells pretreated with 10 μM 4P-PDOT for 1 h, followed by the addition of 1 mM melatonin, and then cultured in growth medium with 120 ng/mL BMP2 for 72 h. (E) Quantification of the western blot in (D). (F) RT-qPCR was used to analyze the expression levels of the Runx2 gene in C3H10T1/2 cells pretreated with 10 μM 4P-PDOT for 1 h, followed by the addition of 1 mM melatonin, and then cultured in growth medium with 120 ng/mL BMP2 for 72 h. (G,H) C3H10T1/2 cells were pretreated with 10 μM 4P-PDOT or luzindole for 1 h, followed by the addition of 1 mM melatonin, and then cultured in growth medium with 120 ng/mL BMP2 for 72 h. (G) ALP and (H) alizarin red staining were performed after 2 weeks or 4 weeks. Data are presented as the mean±SD. **P<0.01. At least three independent experiments were performed. Representative results are shown. Scale bar=100 μm.

5-Fluorouracil (5FU), an antimetabolic drug mainly used in the treatment of tumors, was reported to be able to decrease Runx2 expression [10]. To imitate an unfavorable environment that induces downregulation of Runx2 expression, we added 5FU to C3H10T1/2 cells. These cells were incubated in growth culture medium containing 5FU at different concentrations for 24 h, and then WB analysis and RT-qPCR were used to measure Runx2 expression at the protein and mRNA levels, respectively. We found that Runx2 expression was significantly inhibited by the addition of 5FU in a dose-dependent manner ( Supplementary Figure S3A‒C). Based on the above results, we chose 100 μM 5FU for the subsequent research.

To test whether melatonin prevents 5FU-induced decreased Runx2 expression, C3H10T1/2 cells were pretreated with 1 mM melatonin for 1 h before adding 100 μM 5FU for 24 h in basal culture medium. We found that although the effect of melatonin alone on Runx2 expression was very weak, pretreatment with melatonin could significantly prevent the 5FU-induced decrease in Runx2 expression ( Supplementary Figure S3D‒F). Additionally, to further determine whether melatonin can prevent 5FU-induced inhibition of osteogenic differentiation of MSCs, C3H10T1/2 cells were pretreated with 1 mM melatonin for 1 h before adding 100 μM 5FU for 2 or 4 weeks in osteogenic culture medium. Alkaline phosphatase activity and alizarin red staining showed that although melatonin might promote osteogenic differentiation of MSCs, pretreatment with melatonin could significantly prevent 5FU-induced inhibition of osteogenic differentiation ( Supplementary Figure S3G,H). These results demonstrate that when MSCs are subjected to an inhibited osteogenic stimulus, melatonin plays a synergistic rather than a major role during osteogenic differentiation in mouse mesenchymal stem cell line C3H10T1/2.

To further verify whether the actions of melatonin on 5FU-induced inhibitory effects during osteogenic differentiation of MSCs are associated with MT1 and MT2, C3H10T1/2 cells were pretreated with 10 μM luzindole or 10 μM 4P-PDOT for 1 h before adding 1 mM melatonin, and then 100 μM 5FU was added to the cells for 24 h in growth culture medium or for 2 or 4 weeks in osteogenic culture medium. We found that luzindole or 4P-PDOT could strikingly block the role of melatonin in preventing 5FU-induced inhibition of Runx2 protein and mRNA expressions and osteogenic differentiation of MSCs ( Figure 2). These data indicate that the synergistic role of melatonin in preventing 5FU-induced inhibition of osteogenic differentiation of MSCs is to a great extent via MT2 rather than MT1.

**Figure 2:**
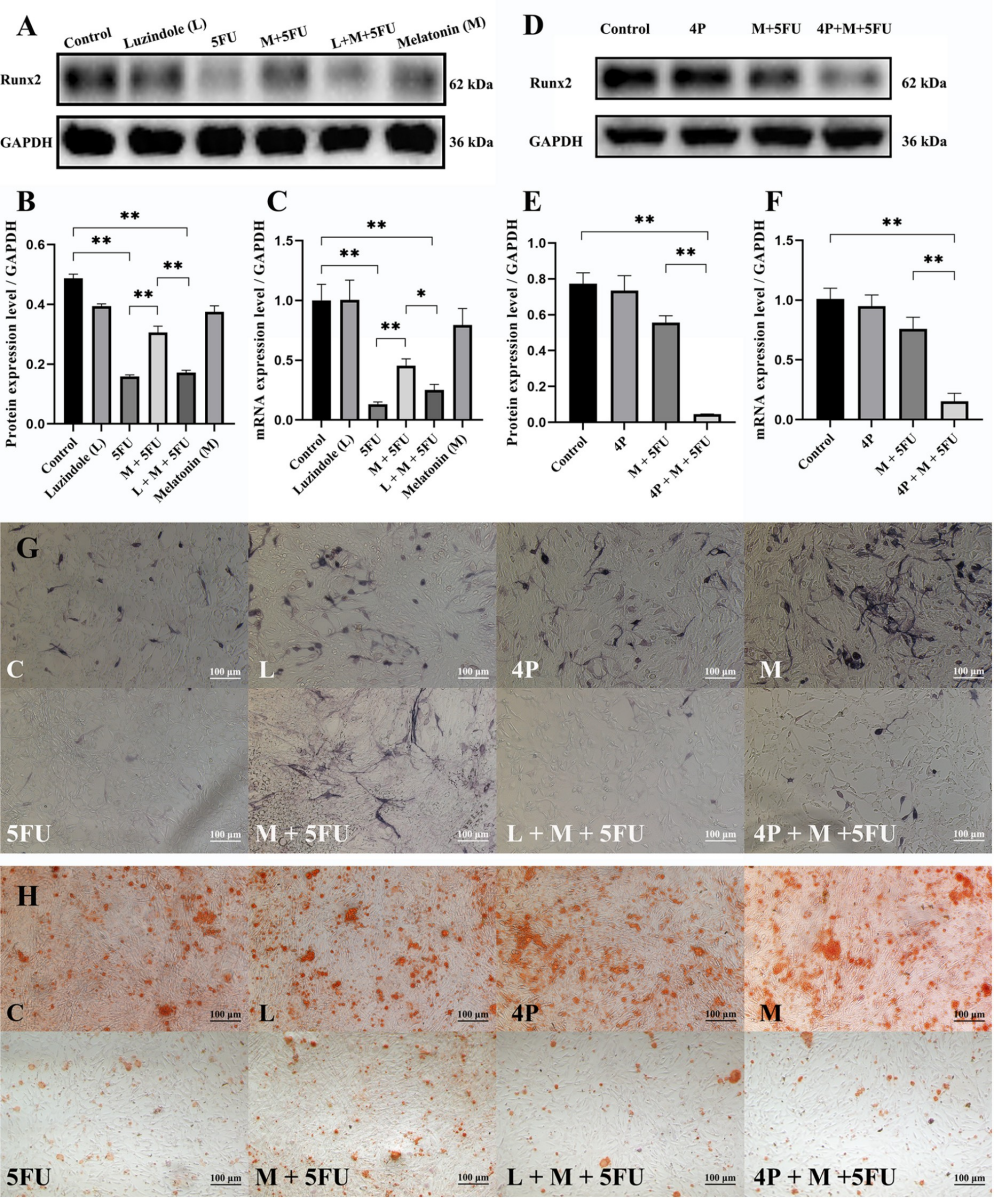
Figure 2 The synergistic effect of melatonin on 5FU-induced inhibited osteogenic differentiation of MSCs is associated with MT1 and MT2 (A) Western blot analysis was used to analyze Runx2 protein expression levels in C3H10T1/2 cells pretreated with 10 μM luzindole for 1 h, followed by the addition of 1 mM melatonin, and then cultured in growth medium with 100 μM 5FU for 24 h. (B) Quantification of the western blot in (A). (C) RT-qPCR was used to analyze the expression levels of the Runx2 gene in C3H10T1/2 cells pretreated with 10 μM luzindole for 1 h, followed by the addition of 1 mM melatonin, and then cultured in growth medium with 100 μM 5FU for 24 h. (D) Western blot analysis was used to analyze Runx2 protein expression levels in C3H10T1/2 cells pretreated with 10 μM 4P-PDOT for 1 h, followed by the addition of 1 mM melatonin, and then cultured in growth medium with 100 μM 5FU for 24 h. (E) Quantification of the western blot in (D). (F) RT-qPCR was used to analyze the expression levels of the Runx2 gene in C3H10T1/2 cells pretreated with 10 μM 4P-PDOT for 1 h, followed by the addition of 1 mM melatonin, and then cultured in growth medium with 100 μM 5FU for 24 h. (G,H) C3H10T1/2 cells were pretreated with 10 μM 4P-PDOT or luzindole for 1 h, followed by the addition of 1 mM melatonin, and then cultured in growth medium with 100 μM 5FU for 24 h. (G) ALP and (H) alizarin red staining were performed after 2 weeks or 4 weeks. Data are presented as the mean±SD. *P<0.05, **P<0.01. At least three independent experiments were performed. Representative results are shown. Scale bar=100 μm.

In conclusion, melatonin plays a synergistic rather than a major role during osteogenic differentiation by upregulating Runx2 expression via MT2 rather than MT1. Our findings provide a new basis and support for further exploring adjuvant treatment of osteoporosis with melatonin.

## Supplementary Data

Supplementary data is available at *Acta Biochimica et Biophysica Sinica* online.

## Supporting information

Supplementary_Fig

Supplementary_Fig

Supplementary_Fig
