# Supplementary figures and images for: Melatonin plays a synergistic rather than a major role during osteogenic differentiation via MT2 in mouse mesenchymal stem cell line C3H10T1/2: Melatonin plays a synergistic role during osteogenic differentiation
Source: Acta Biochim Biophys Sin (Shanghai). 2023 Jul 5;55(9):1506–10. doi: 10.3724/abbs.2023095 (PMC10520480; doi:10.3724/abbs.2023095)

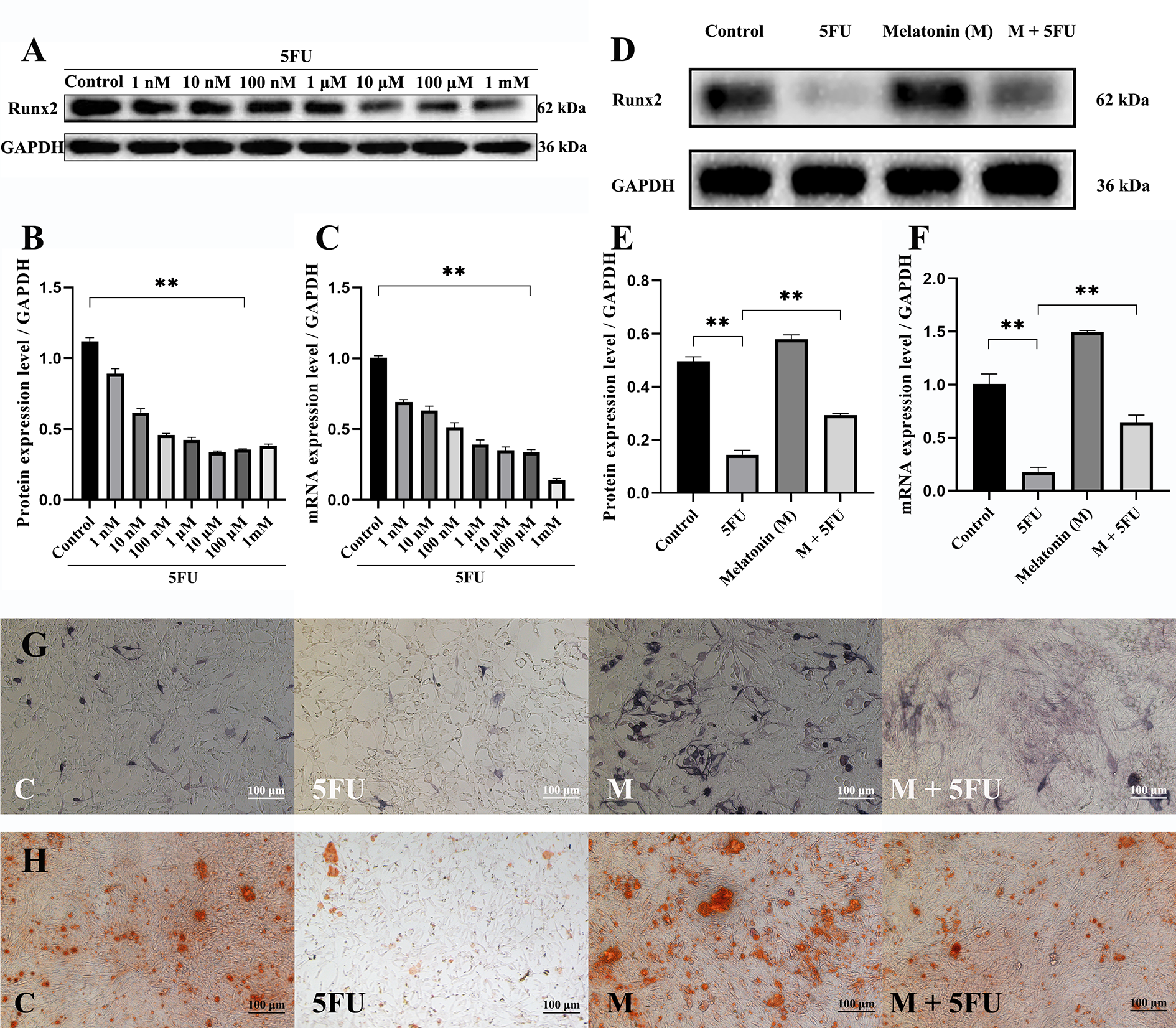

Supplement: Supplementary_Fig [file Supplementary_Fig._3.tif]

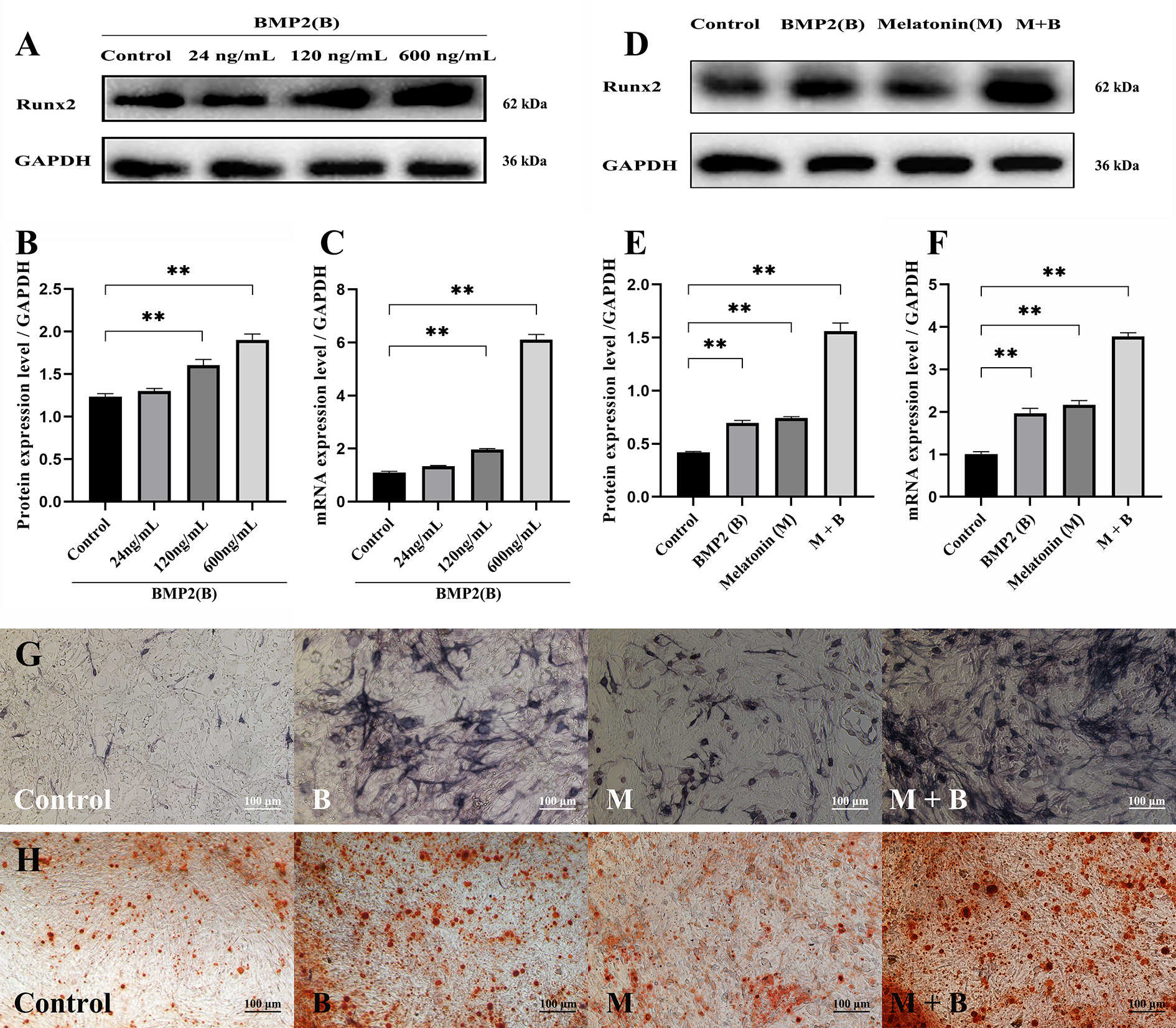

Supplement: Supplementary_Fig [file Supplementary_Fig._2.tif]

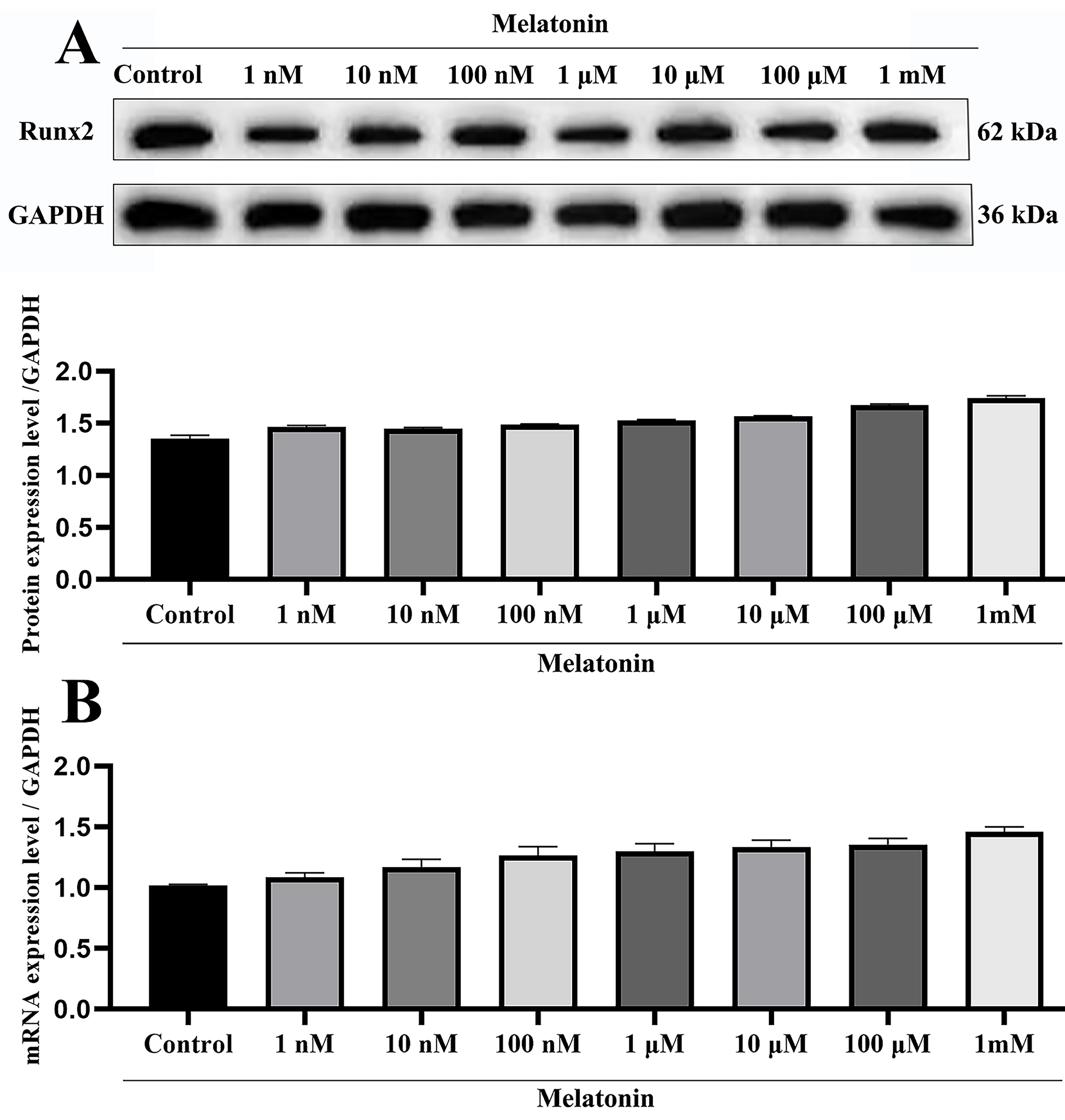

Supplement: Supplementary_Fig [file Supplementary_Fig._1.tif]
